# Influence on Secondary Metabolism of *Piper nigrum* L. by Co-Inoculation with Arbuscular Mycorrhizal Fungi and *Fusarium solani* f. sp. *piperis*

**DOI:** 10.3390/microorganisms9030484

**Published:** 2021-02-25

**Authors:** Rafaela Trindade, Laís Almeida, Luciana Xavier, Eloisa Helena Andrade, José Guilherme Maia, Andréa Mello, William N. Setzer, Alessandra Ramos, Joyce Kelly R. da Silva

**Affiliations:** 1 Programa de Pós-Graduação em Biotecnologia, Universidade Federal do Pará, Belém 66075-110, Brazil; rafacabral.bio@gmail.com (R.T.); laisalmeida00@hotmail.com (L.A.); 2 Laboratório de Biotecnologia de Enzimas e Biotransformações, Universidade Federal do Pará, Belém 66075-110, Brazil; lpxavier@ufpa.br; 3 Coordenação de Botânica, Museu Paraense Emílio Goeldi, Belém 66040-170, Brazil; eloisa@museu-goeldi.br; 4 Departamento de Química, Universidade Federal do Maranhão, São Luís 65080-805, Brazil; gmaia@ufpa.br; 5 Instituto de Estudos de Desenvolvimento Agrário Regional, Universidade Federal do Sul e Sudeste do Pará, Marabá 68507-590, Brazil; andreahentz@unifesspa.edu.br; 6 Department of Chemistry, University of Alabama in Huntsville, Huntsville, AL 35899, USA; wsetzer@chemistry.uah.edu; 7 Aromatic Plant Research Center, 230 N 1200 E, Suite 100, Lehi, UT 84043, USA; 8 Instituto de Estudos em Saúde e Biológicas, Universidade Federal do Sul e Sudeste do Pará, Marabá 68507-590, Brazil; rezende@unifesspa.edu.br

**Keywords:** black pepper, fusariosis, mycorrhiza, β-caryophyllene, 2*E*-hexenal, sesquiterpene hydrocarbons, oxygenated sesquiterpenes

## Abstract

To elucidate defense mechanisms of *Piper nigrum* against fusariosis, an experiment based on co-inoculation of arbuscular mycorrhizal fungi (AMF) and *Fusarium solani* f. sp. *piperis* was performed. Variations in secondary metabolism in plants infected with *F. solani* f. sp. *piperis* (FUS) and co-inoculated with AMFs and *F. solani* (AMF + FUS) were monitored at 7- and 21-days post inoculations (dpi). The pathogen induced a decrease in oxygenated sesquiterpenes (82.0–77.4%), and changes in the concentrations of the main compounds, α-muurolene, α-muurolol, and 2*E*-hexenal in the leaves. It was observed that the concentration of 2*E*-hexenal decreased at 7 dpi, α-muurolene decreased at 21 dpi, and α-muurolol increased at 21 dpi. There was a prevalence of sesquiterpene and monoterpene hydrocarbons in the roots, such as β-caryophyllene, δ-elemene, and limonene. The infection and co-inoculation induced greater production of phenolics in the roots at 7 dpi. The enzymatic activity of phenylalanine ammonia-lyase decreased in the leaves at 21 dpi and in the roots on both days, while the lipoxygenase activity decreased only in the roots at 21 dpi. The results demonstrated that co-inoculation with AMFs and *F. solani* induces changes in the defense metabolism of *P. nigrum*, but it is not efficient in the biocontrol of fusariosis during the evaluated period.

## 1. Introduction

Black pepper fusariosis, root rot, foot rot, or ‘mal de mariquita’ is caused by *Fusarium solani* f. (Mart.) Sac. f. sp. Albuq *piperis* or *Nectria haematococca* f. sp. *piperis*, teleomorphic form. It is a phytopathology of great economic interest for the state of Pará, Brazil, and represents the main problem for the cultivation of black pepper, as it has decimated large planted areas and culminated in the increase in production and sale costs of black pepper fruit [1,2].

Fusariosis is established through a compatible interaction between the host and the pathogen [3]. Some *Piper nigrum* cultivars are more susceptible than others, the Bragantina cultivar for instance, despite its high yield in fruit production, is one of the most vulnerable to disease [3,4]. The main form of disease control in the field is the use of cultural practices and fungicide application, as well as the production of resistant cultivars [2,5]. However, the genetic vulnerability of Amazonian cultivars and the absence of fusariosis in India, the *P. nigrum* diversity center, constitute the main limitations for the genetic improvement of black pepper [6].

Biological control of soil-borne pathogens is an environmentally friendly, feasible, and economical alternative to the use of chemical fungicides [7]. *Trichoderma* species and endophytic bacteria (*Bacillus* sp., *Enterobacter* sp., *Rhizobium* sp.) were effective in controlling black pepper pathogenic fungi [8]. Studies with arbuscular mycorrhizal fungi (AMF) showed that *Scutellospora gilmorei* was efficient in controlling black pepper fusariosis [9], in addition to improving biomass and defense metabolism of *P. nigrum* [10,11].

The mechanisms by which AMFs control pathogens are not yet fully known and generally depend directly on the species used [9,12]. However, the main propositions are that AMF colonization results in changes in the root architecture, morphology, and chemical composition of the root exudates, which may be responsible for altering the dynamics of the infection [13]. In addition, symbiosis with AMFs can induce signaling and resistance mechanisms in the plant due to changes in secondary metabolism and expression of defense-related genes, with the production of enzymes of the phenylpropanoid and lipoxygenase pathways, chitinases, β-1,3-glucanases, peroxidases, pathogenesis-related proteins, and hydroxyproline-rich glycoproteins [14,15,16]. In regard to volatile organic compounds (VOCs), MFAs can induce changes in the concentration and composition of terpenoids, increasing plant defenses against pathogens [17,18].

Thus, due to the existence of very few studies relating the influence of beneficial microorganisms in the control of fusariosis and the lack of knowledge of the profile of volatiles emitted by *Piper nigrum* as an initial response to co-inoculation with symbiotic and pathogenic microorganisms, the present study aimed to assess changes in secondary metabolism of black pepper seedlings caused by co-inoculation with AMFs and *F. solani* f. sp. *piperis*.

## 2. Materials and Methods

### 2.1. Plant Material

Seedlings of *Piper nigrum* L. cv. Bragantina with approximately three months of age were provided by Empresa Brasileira de Pesquisa Agropecuária-Amazônia Oriental (EMBRAPA). The seedlings were acclimatized in a greenhouse, located at the Instituto de Ciências Biológicas of Universidade Federal do Pará, under lateral and upper shading of 50% and 70%, respectively; watered daily according to the soil field capacity. Thirty days later, the seedlings were transplanted, one plant per pot, using a commercial substrate made up of a mixture of expanded vermiculite type B, limestone, bone meal, and castor bean (*Ricinus communis*). The *P. nigrum* was vouchered (voucher number MG224384) with the Herbarium of Museu Paraense Emílio Goeldi (MPEG).

### 2.2. Preparation of Arbuscular Mycorrhizal Fungal Spore Inoculum and Inoculation

The species of the arbuscular mycorrhizal fungi *Rhizophagus clarus* and *Claroideoglomus etunicatum* were collected from rhizospheric soil samples from southeast of Pará state (Amazon region, Brazil). The spores were cultivated and multiplied in sterile sand, using *Brachiaria brizantha* (A. Rich.) Stapf as a trap culture, under greenhouse conditions. The identification of the species was performed by morphological comparison based on the manual INVAM (International Collection of Cultures of Arbuscular Mycorrhizal (Vesicular) Fungi). Inoculum was prepared with the proportion of 50% of each species of fungus, composed of a mixture of spores (density of 90 spores/g soil), hyphae, root fragments, and sterile sand. The seedlings were removed from the planting bags and pits approximately 2 cm deep were opened, 6-g inocula containing AMF spores were dispersed superficially on the roots, which were finally covered by the substrate.

### 2.3. Acquisition, Isolation, and Culture of Fusarium solani f. sp. piperis Phytopathogen

The strain *Fusarium solani* f. sp. *piperis* was provided by EMBRAPA, which was isolated from plants presenting symptoms of fusariosis cultivated in Bujarú City (PA, Brazil). The pathogen was cultivated in sterile Petri dishes containing potato dextrose agar (PDA) at room temperature and identified based on the characteristics of its macroscopic colonies, hyphae, and conidia.

### 2.4. Experimental Design and Material Collection

**Figure 1:**
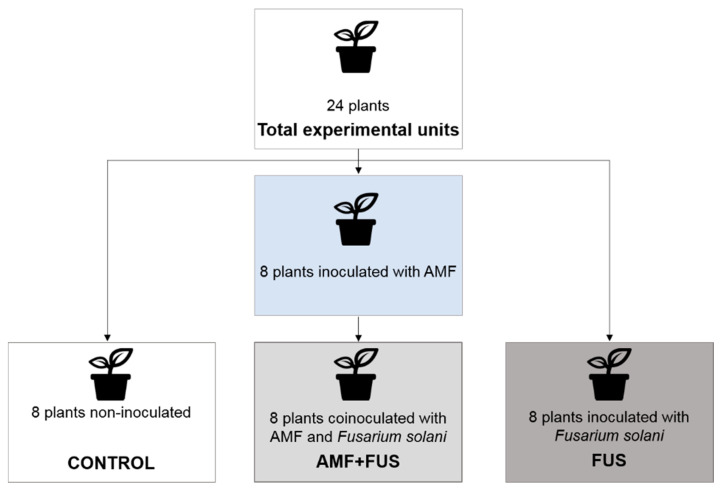
Diagram of the experimental design, groups that were used in the experiments.

Twenty-four seedlings were used in the experiment and have been separated into CONTROL group (not inoculated), FUS (plants inoculated with *F. solani* f. sp. *piperis*), and AMF + FUS (plants co-inoculated with arbuscular mycorrhizal fungi and *F. solani* f. sp. *piperis*) (Figure 1). After the inoculations, the plants were separated and randomly arranged in the greenhouse to avoid contamination among the treatments. Samples of leaves and roots of each seedling were collected at 7- and 21-days post inoculation (dpi).

### 2.5. Inoculation, Observation of Symptoms and Recovery of Fusarium solani f. sp. piperis

After 15 days of growth, 10 mL of sterile water was added to each of the Petri dishes. Hyphae that contained conidia were removed using a spatula, to give a final suspension volume of 1.5 L. The concentration of spores in the suspension was determined microscopically using a Neubauer chamber and found to be 1.12 × 10^6^ mL^−1^ [19]. Twenty days after the inoculation of AMFs, a total of 16 plants were inoculated with *F. solani* f. sp. *piperis*, to compose the AFM + FUS and FUS groups, according to the methodology described by da Luz et al. (2017) [20]. After being removed from the polypropylene bags, their roots were washed and immersed for five seconds in the conidial suspension. At the end, the seedlings were replanted and kept in the greenhouse for 21 days, during which the symptoms were monitored. The seedlings of the control group were not inoculated. After inoculation with *F. solani* f. sp. *piperis* the symptoms of the aerial part and roots of the seedlings were monitored. After 21 days of infection, the pathogen was again isolated from the seedlings, small pieces of tissue collected near the symptomatic locations were sterilized with water, alcohol 70% and 10% sodium hypochlorite for one minute, they were placed in contact with PDA medium at 27 °C and after 10 days of incubation the fungal culture was isolated [19].

### 2.6. Extraction and Volatile Compounds

The leaf and root essential oils were obtained from fresh *P. nigrum* plant material (2.0 g) using a Likens–Nickerson apparatus with simultaneous extraction with *n*-pentane (3 mL) over a period of 2 h. For each essential oil extracted, a 1.0-mL aliquot (1.0 μL) was analyzed using gas chromatography–mass spectrometry (GC-MS) as previously described (da Trindade et al., Plants, 2019, 8(11), 442): Shimadzu QP2010 plus instrument; Rtx-5MS capillary column (30 m × 0.25 mm × 0.25 mm film thickness); temperature ramp of 60–240 °C at a rate of 3 °C/min; injector temperature of 200 °C; He carrier gas with 1.2 mL/min flow rate; split mode injection (20:1 split ratio); MS ionization voltage = 70 eV; ion source and detector temperature = 200 °C. Compound identification was achieved by comparison of the retention indices, determined using a homologous series of *n*-alkanes (C8–C32, Sigma–Aldrich, St. Louis, MO, USA) [21], and the mass spectral fragmentation patterns with those reported in the databases [22,23,24].

### 2.7. Lipoxygenase (LOX) Activity

The roots and leaves were macerated in liquid nitrogen. For each sample, pulverized plant material (1.0 g) was mixed with 3 mL of sodium phosphate buffer (50 mM, pH 6.5) and the mixture centrifuged. The supernatant so obtained was used as the enzyme source. A sodium linoleate solution was prepared by mixing linoleic acid (78 μL, Sigma-Aldrich, St. Louis, MO, USA) and Tween 20 (90 μL, Sigma-Aldrich) with boiling water (10 mL) and then adding a few drops of sodium hydroxide (0.5 N). The final linoleate solution was adjusted to 25 mL, to give a 10 mM sodium linoleate solution, which was stored at −20 °C. Determination of LOX activity was carried out using 5 μL of the enzyme solution, 50 μL of sodium linoleate (10 mM) solution, and diluted with 1945 μL of sodium phosphate buffer. The formation of the final product was monitored at 234 nm using a UV–visible spectrophotometer. The increase in absorbance at 234 nm indicated the formation of a conjugated double-bond system in the hydroperoxide product [3].

### 2.8. Phenylalanine Ammonia Lyase (PAL) Activity

A homogenous mixture of pulverized roots and leaves (1.0 g), 2.0 mL sodium borate buffer (0.3 mM, pH 8.8), 1 mM dithiothreitol (DTT), 1 mM ethylenediaminetetraacetic acid (EDTA), and 5% polyvinylpolypyrrolidone (PVP) was prepared and centrifuged. A 0.5-mL aliquot of the supernatant was then added to 1.0 mL of reaction buffer that contained sodium borate (0.3 mM, pH 8.8) and L-phenylalanine (0.03 mM). The reaction mixture was incubated for 15 min at room temperature, after which the absorbance at 290 nm was obtained using a UV–visible spectrophotometer. PAL activity was assessed by measuring the concentration of (*E*)-cinnamic acid produced from the L-phenylalanine substrate [25].

### 2.9. Total Phenolics Determination

The fresh leaves and roots (2 g) were extracted with ethyl acetate (50 mL) by percolation at room temperature over a 96-h period. The solvent was evaporated and the total phenolics concentration was determined using the Folin–Ciocalteu method [26]. A 500-μL aliquot of the plant extract was dissolved in methanol (20 mg/mL) and allowed to react with 250 μL of Folin–Ciocalteu reagent (1 N) and 1250 μL of sodium carbonate (75 g/L). The reaction mixture was incubated in the dark for 30 min, after which the absorbance was measured at 760 nm using a UV–visible spectrophotometer. A calibration curve was prepared using gallic acid at concentrations of 0.5 to 10.0 mg L^−1^. The total phenolics concentrations in the *P. nigrum* extracts were then determined as gallic acid equivalents (GAE) in milligrams per gram of extract (mg/GAE g^−1^).

### 2.10. Statistical Analyses

All measurements were carried out in triplicate and compared with the control groups. The data have been expressed as means ± standard deviation. Analysis of variance was conducted by two-way ANOVA followed by the Tukey test using GraphPad 7.0 software. Differences at *p* < 0.05 were considered significant. To verify the similarity between the groups in the experiment, a hierarchical cluster analysis (HCA) was performed using the Euclidean distance and complete linkage methods. The analysis was done with data from classes of volatile, phenolic compounds, LOX, and PAL for leaves and roots. Data were analyzed using the Minitab 19 software (free version, Minitab software, State College, PA, USA).

## 3. Results

### 3.1. Aspects of Infection

Visual symptoms of fusariosis were monitored to evaluate the development of the disease. In the leaves, chlorosis was noticed from 15 dpi on inoculated plants (FUS) and co-inoculated with AMFs and *F. solani* f. sp. *piperis* (AMF + FUS), which culminated in the premature fall of some leaves. These symptoms were absent in the control seedlings. On the last day of collection (21 dpi), the inoculated plant roots (FUS) showed a dark color and necrosis along their length, characterizing root rot. The same appearance was noticed in the co-inoculated plants (AMF + FUS), however with less intensity.

### 3.2. Oil Essential Composition

**Figure 2:**
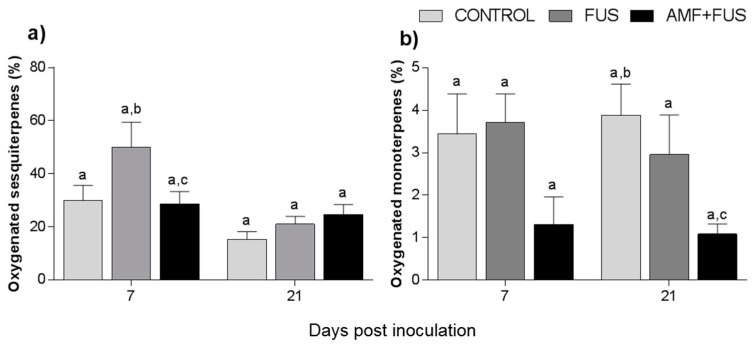
Variation of the terpene content of *Piper nigrum* L. leaves (CONTROL) after inoculation with *Fusarium solani* f. sp. *piperis* (FUS) and co-inoculation with arbuscular mycorrhizal fungi (AMF + FUS). (**a**) leaves; (**b**) roots. Different letters indicate statistical variation by the Tukey test (*p* < 0.05).

Variations in volatile compounds caused by inoculation with the pathogen and co-inoculation with AMFs and *F. solani* were evaluated throughout the study. A total of 71 volatile compounds were identified in the leaves, corresponding to 97.3% of total composition. The CONTROL, FUS, and AMF + FUS groups were rich in sesquiterpene hydrocarbons at 7 dpi (45.0, 78.86, and 55.25%) and at 21 dpi (72.92, 51.93, and 55.20%), respectively; as well as in oxygenated sesquiterpenes at 7 dpi (29.96, 50.04, and 28.64%) and 21 dpi (15.35, 21.69, and 24.3%), respectively. There was variation in oxygenated sesquiterpenes in the leaves at 7 dpi, which were lower in the AMF + FUS (28.64%) compared to the FUS group (50.04%) (Figure 2a, Table 1).

**Figure 3:**
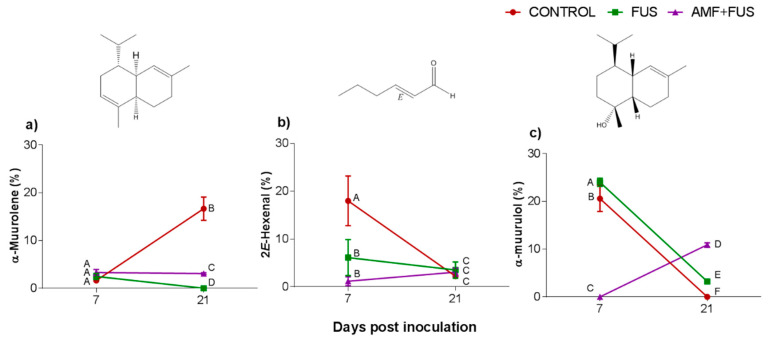
Variation of the major volatile compounds of *Piper nigrum* L. (CONTROL) leaves after inoculation with *Fusarium solani* f. sp. *piperis* (FUS) and co-inoculation with arbuscular mycorrhizal fungi and *F. solani* f. sp. *piperis* (AMF + FUS). (**a**) α-muurolene; (**b**) 2*E*-hexenal; (**c**) α-muurulol. Different letters mean significant statistical difference by Tukey’s test (*p* < 0.05).

The major compounds in the leaves were α-muurolene, 2*E*-hexenal, and α-muurolol (Figure 3, Table 1). The amount of α-muurolene was lower at 21 dpi, FUS (16.68–0.0%) and AMF + FUS (16.68–3.08%), thus the compound was greater in the AMF + FUS that FUS group. At 7 dpi, 2*E*-hexenal content in FUS (6.11%) and AMF + FUS (1.16%) was lower in relation to the CONTROL group (17.98%). 2*E*-hexenal aldehyde was produced in *P. nigrum* leaves in all evaluated treatments. At 7 dpi FUS produced a higher amount of α-muurolol (24.0%) compared to the CONTROL (20.59%) and AMF + FUS (0.0%) groups, at 21 dpi, both FUS (3.0%) and AMF + FUS (10.91%) treatments produced a higher amount of α-muurolol than the CONTROL group (0.0%).

**Figure 4:**
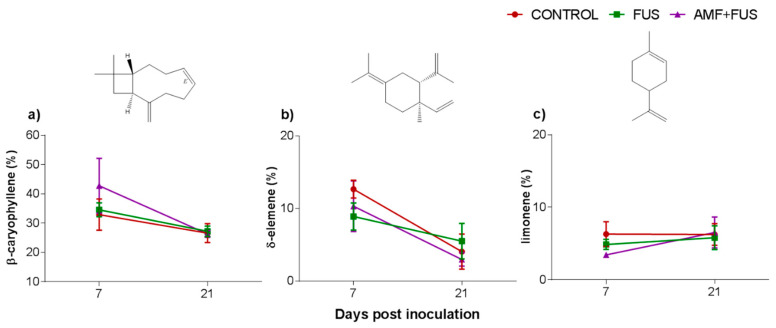
Major volatile compounds of roots of *Piper nigrum* L. (CONTROL) after inoculation with *Fusarium solani* f. sp. *piperis* (FUS) and co-inoculation with arbuscular mycorrhizal fungi and *F. solani* f. sp. *piperis* (AMF + FUS). (**a**) β-caryophyllene; (**b**) δ-elemene; (**c**) limonene.

A total of 37 volatile compounds were identified in the roots, corresponding to 72% of total composition. The CONTROL, FUS, and AMF + FUS groups were rich in monoterpene hydrocarbons at 7 dpi (15.37, 15.74, and 15.37%) and at 21 dpi (22.63, 21.1, and 19.75%), respectively; as well as in sesquiterpene hydrocarbons at 7 dpi (51.8, 49.1, and 51.80%) and 21 dpi (35.37, 37.07, and 32.91%), respectively. The oxygenated monoterpenes were lower in AMF + FUS (1.08%) compared to CONTROL (3.88%) at 21 dpi (Figure 2b, Table 1). The major volatiles compounds found in roots were β-caryophyllene, δ-elemene, and limonene (Figure 4, Table 2).

### 3.3. Total Phenolic Content

**Figure 5:**
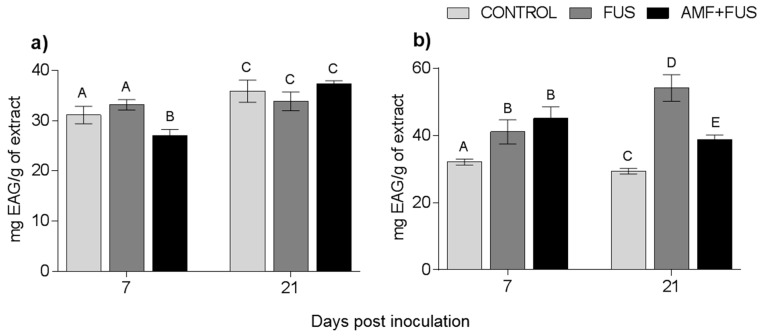
Variation of phenolic compounds produced by *Piper nigrum* L. (CONTROL) seedlings after inoculation with *Fusarium solani* f. sp. *piperis* (FUS) and co-inoculation with arbuscular mycorrhizal fungi and *F. solani* f. sp. *piperis* (AMF + FUS). (**a**) leaves; (**b**) roots. Different letters varied statistically by Tukey’s test (*p* < 0.05).

Changes in the content of phenolic compounds were measured after the inoculation and co-inoculation of *P. nigrum*. The total phenolic content of *P. nigrum* leaves at 7 dpi decrease in AMF + FUS (27.06 mg EAG/g) compared with CONTROL (31.12 EAG/g) and FUS groups (33.15 mg EAG/g), and FUS was superior to AMF + FUS (33.15, 27.06 mg EAG/g) (Figure 5a). In roots, there were variations in all days analyzed, at 7 dpi both FUS (41.06 EAG/g) and AMF + FUS (45.08 mg EAG/g) were higher than CONTROL (32.07 mg EAG/g). At 21 dpi, FUS (54.14 mg EAG/g) and AMF + FUS (38.78 mg EAG/g) produced higher phenolic content than CONTROL plants (29.38 mg EAG/g). When comparing the two treatments, FUS (54.14 mg EAG/g) was greater than AMF + FUS (38.78 mg EAG/g) (Figure 5b).

### 3.4. Enzymatic Activity

#### 3.4.1. Lipoxygenase Enzyme

**Figure 6:**
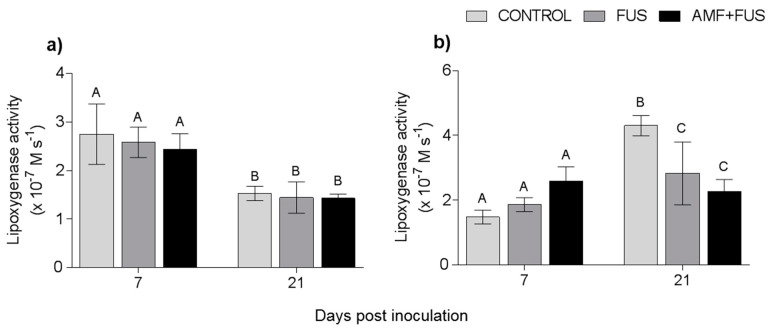
Variation in lipoxygenase activity in *Piper nigrum* L. (CONTROL) seedlings after inoculation with *Fusarium solani* f. sp. *piperis* (FUS) and co-inoculation with arbuscular mycorrhizal fungi and *F. solani* f. sp. *piperis* (AMF + FUS). (**a**) leaves; (**b**) roots. Different letters varied statistically by Tukey’s test (*p* < 0.05).

Variations in lipoxygenase activity were assessed during the study. LOX activity was unchanged in leaves, however in the roots the FUS (2.83 × 10^−7^ M.s^−1^) and AMF + FUS (2.26 × 10^−7^ M.s^−1^) were lower than the CONTROL (4.3 × 10^−7^ M.s^−1^) at 21 dpi (Figure 6).

#### 3.4.2. Phenylalanine Ammonia-Lyase Enzymatic Activity

**Figure 7:**
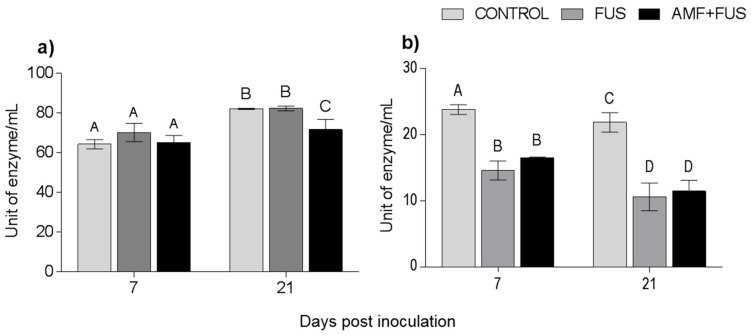
Phenylalanine ammonia-lyase enzyme activity in *Piper nigrum* L. (CONTROL), inoculated with *Fusarium solani* f. sp. *piperis* (FUS) and co-inoculated with arbuscular mycorrhizal fungi and *F. solani* (AMF + FUS). (**a**) leaves; (**b**) roots. Different letters varied statistically by Tukey’s test (*p* < 0.05).

During the experiment, the enzymatic activity of phenylalanine ammonia-lyase was quantified. There were variations in the PAL activity in leaves and roots (Figure 7). At 21 dpi, AMF + FUS plants show lower activity in leaves (71.43 unit of enzyme/mL [U/mL]) compared to CONTROL group (81.91 U/mL) and FUS (82.16 U/mL). In roots at 7 dpi, both the FUS (14.06 U/mL) and AMF + FUS (16.55 U/mL) were lower than the CONTROL group (23.08 U/mL), and at 21 dpi, FUS (10.59 U/mL) and AMF + FUS (11.47 U/mL) were lower than the CONTROL group (21.86 U/mL).

### 3.5. Hierarchical Cluster Analysis

**Figure 8:**
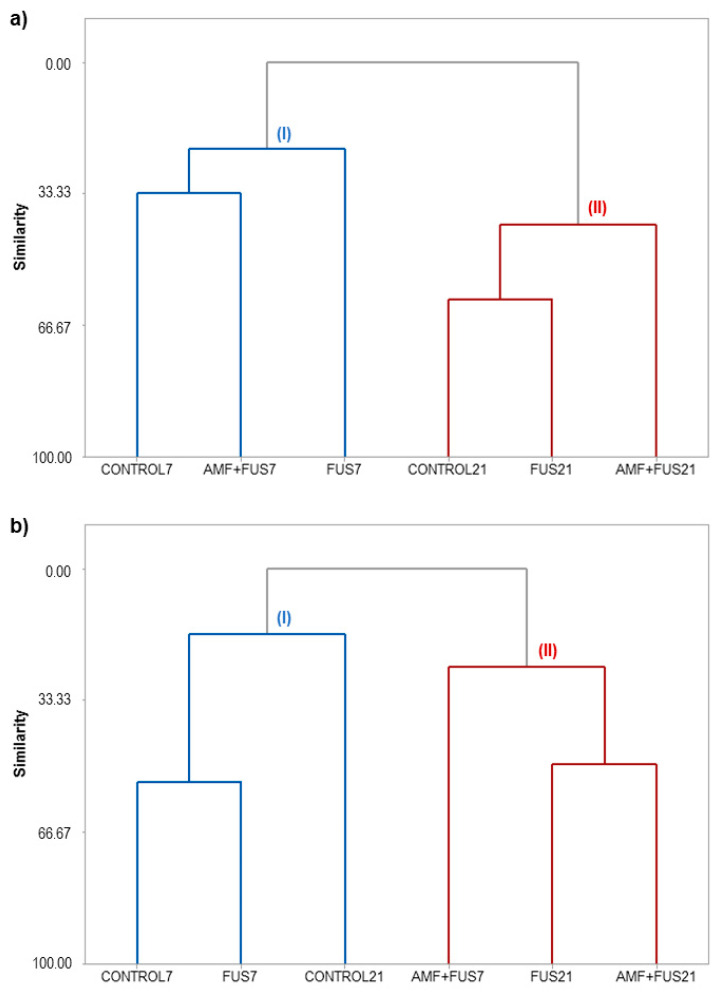
Hierarchical clusters analysis (HCA) of *P. nigrum* Bragantina cv. at 7- and 21-days post inoculations based on the classes of volatile, phenolic compounds, lipoxygenase, and phenylalanine ammonia-lyase enzymes. *P. nigrum* seedlings: CONTROL7, CONTROL21; *P. nigrum* inoculated with *Fusarium solani* f. sp. *piperis*: FUS7, FUS21; *P. nigrum* co-inoculated with AMFs and *F. solani* f. sp. *piperis*: AMF + FUS7, AMF + FUS21. (**a**) leaves; (**b**) roots.

The similarity levels among the CONTROL, FUS, and AMF + FUS treatments for leaves and roots at 7 and 21 dpi can be visualized in the dendrograms (Figure 8).

HCA analysis of the leaves grouped the samples into clusters I and II (Figure 8a). Cluster I was composed of the samples CONTROL7, FUS7, and AMF + FUS7, and cluster II was grouped with CONTROL21, FUS21, and AMF + FUS21. The co-inoculation (AMF + FUS7) maintained the volatile contents similar to CONTROL7 group; there was also a balance in the activity of defense enzymes and phenolic compounds. On the other hand, seedlings infected by the pathogen (FUS7) showed a secondary metabolism composition different from the other treatments. At 21 dpi, when the first symptoms appear, co-inoculation (AMF + FUS21) showed a different profile from the control and infected plants (FUS21). In co-inoculated plants, there was a decrease in the α-muurolene content (3.08%) compared to the control group (16.68%) and lower activity of PAL (71.42 U/mL) compared to control (81.91 U/mL) and infected plants (82.16 U/mL). On the other hand, the inoculated group was closer to the control group. The dendrogram obtained for the roots classified the samples into two clusters (Figure 8b). Cluster I grouped the samples CONTROL7, FUS7, and CONTROL21, and cluster II was composed of the samples AMF + FUS7, FUS21, and AMF + FUS21. The infection by *Fusarium* (FUS7) maintained the volatile contents similar to the control group at 7 and 21 dpi. However, the secondary metabolites in co-inoculated plants (AMF+ FUS21 and AMF + FUS7) were similar to the infected group (FUS21) forming the cluster II.

## 4. Discussion

Co-inoculation of AMFs and the pathogen *F. solani* f. sp. *piperis* in *P. nigrum* Bragantina cv. promoted variations in the volatile contents, phenolic compounds, and in the activity of enzymes related to plant defense.

During mycorrhizal colonization, there is usually a greater allocation of carbon to the plant’s root system to enable and maintain symbiosis, thus less carbon content is available for photosynthesis and to produce plant secondary compounds. In the early stages of mycorrhizal colonization, the production of plant defense compounds is modulated by AMFs, to allow its establishment in the plant tissue, so there may be a reduction in the production of plant defense compounds. On the other hand, in the more advanced stages of the association, strong activation of the plant defense system can occur [27,28]. A study was carried out on the inoculation of AMFs in *P. nigrum* Bragantina cv. and evaluated the production of volatile compounds for 60 days. At 7 dpi the symbiosis was already fully established with the presence of hyphae, arbuscules, and vesicles. Regarding the major volatile compounds, they were the same over 60 days, which varied in quantity, some increased and others decreased in content from 7 dpi. Considering these aspects, in the present study, after inoculation of *P. nigrum* seedlings with AMFs, 20 days were allowed to elapse prior to co-inoculate the AMF + FUS group with *F. solani*. The experiment was conducted in this way to ensure the establishment of symbiosis and ‘normalize’ the plant’s defense metabolism [11].

Aspects of plant-pathogen (FUS) interaction and co-inoculated seedlings (AMF + FUS) were monitored visually during the experiment period. Leaf yellowing was observed at 15 dpi in inoculated (FUS) and co-inoculated plants (AMF + FUS), however they were absent in the control seedlings. The first symptoms of fusariosis in *P. nigrum* Bragantina cv. can appear during the first weeks after inoculation, some studies have noticed chlorosis and leaf wilt from 21 dpi [20,29]. Symptoms of fusariosis can vary according to the susceptibility of *P. nigrum* cultivars, but they usually start in the secondary roots with progressive rotting of the root system, leading to yellowing and withering of the leaves, which can fall or necrotize [3].

*Piper nigrum* seedlings evaluated in this study showed the predominance of terpenoids as sesquiterpene hydrocarbons (45.9%) and oxygenated sesquiterpenoids (29.96%) in the leaves, and sesquiterpene and monoterpene hydrocarbons (51.8%, 15.37%) in the roots. Similarly, it was verified that *P. nigrum* cv. Bragantina seedlings synthesized mostly sesquiterpene hydrocarbons (25.0%) and oxygenated sesquiterpenoids (67.0%) in the leaves [4], and sesquiterpene and monoterpene hydrocarbons (62.95%, 25.89%) in the roots [11]. The *F. solani* f. sp. *piperis* inoculation induced an increase in sesquiterpene hydrocarbons (45.9–78.86%) at 7 dpi, likewise *P. nigrum* cv. Bragantina leaves showed an increase in sesquiterpene hydrocarbons (3.7–10.9%) at 7 dpi after the inoculation with this phytopathogen [20]. An increase of oxygenated sesquiterpenes was observed in leaves from plants inoculated with the pathogen (45.09–78.86%), however there was a decrease in this volatile class after the co-inoculation with AMFs and *F. solani* (78.86–56.25%).

At 7 dpi of experiment, 2*E*-hexenal concentration decreased in the leaves of FUS (6.11%) and AMF + FUS (1.16%) compared to the CONTROL (17.98%) group. It has been shown that (*E*)-2-hexenal activated the jasmonic acid (JA) pathway in *Arabidopsis*, improving resistance against the necrotrophic fungus *Botrytis cinerea*, but promoted susceptibility to *Pseudomonas syringae* pv. Tomato [30], the compound may induce defense against some pathogens and circumstantially confer susceptibility to other pathogens. *Fusarium solani* f. sp. *piperis* has a hemibiotrophic lifestyle [31] and establishes an interaction compatible with *Piper nigrum* [20], it may have depressed the defense system of the plant, culminating in the decreased of 2*E*-hexanal at 7 dpi. The 2*E*-hexenal, 2*E*-hexen-1-ol, 3*Z*-hexen-1-ol and 1-hexanol compounds in chickpea (*Cicer arietinum* L.) and wheat (*Triticum* sp.) completely inhibited the growth in vitro of *Fusarium avenaceum* and *F. graminearum*, suggesting that although produced in low concentration in wheat leaves, it may confer resistance against some *Fusarium* species [32]. 2*E*-Hexenal is a volatile organic compound (VOC) produced by JA pathway, in which the lipoxygenase enzyme plays a key role in the production of these volatiles. VOCs are emitted by all plants, both constitutively and in response to biotic and abiotic stress, and are considered mediating signals of communication between plants and other organisms [33]. Biotrophic pathogens can suppress the JA pathway by activating salicylic acid signaling [30]. Thus, the inoculation of AMF in *P. nigrum* may have influenced the decrease of 2*E*-hexenal, as was observed in the leaf’s volatiles.

β-Caryophyllene was one of the major compounds produced in the roots of *P. nigrum*. The restoring of β-caryophyllene in maize (*Zea mays* L.) improved plant resistance against herbivores, but increased susceptibility to infection and growth of the hemibiotrophic fungus *Colletotrichum graminicola*, the stimulatory effect of β-caryophyllene on the fungus is in striking contrast to the numerous studies on the antimicrobial properties of terpenoid volatile compounds [34]. One study showed the potential of *F. solani* in upregulation of the gene encoding the sesquiterpene synthetase enzyme, a *Fusarium solani* mycelium disc was placed on top of the *Piper betle* explants in tissue culture, and a high production of β-cubebene, β-caryophyllene, and germacrene D sesquiterpenes was verified, *P. betle* extract is already known to have allelochemical properties that protect the plant from pathogens and herbivores [35].

When comparing the production of phenolics between leaves and roots, we noticed a higher production and variation in the roots. This may have occurred because the root is the site of infection; there was increase in the phenolic content of roots at 7 and 21 dpi in FUS (41.06, 54.14 EAG/g) and AMF + FUS (45.08, 38.78 EAG/g) in relation to CONTROL (32.07, 29.38 EAG/g), respectively. After co-inoculation of *Bradyrhizobium* sp. and *Glomus mosseae* in soybean seedlings (*Glycine max*), their roots released a larger amount of exudates containing phenolic acids, which significantly reduced the growth and reproduction of the pathogenic fungus *Cylindrocladium parasiticum* [36]. Two cultivars of *Piper nigrum*, Bragantina (susceptible), and Cingapura (tolerant) were inoculated with *F. solani* f. sp. *piperis* and monitored for 45 days; the leaves of Bragantina cv. showed no variation in phenolic content, while the leaves of Cingapura cv. presented high production in inoculated plants only at 7 dpi; the inoculated roots of Bragantina cv. produced less content of total phenolic at 45 dpi compared with control plants, while roots of Cingapura cv. produced higher phenolics content in the inoculated plants at 15, 30, and 45 dpi [20]. Similar to the present study, the roots showed greater variation in phenolic content in relation to the leaves, possibly due to infection occurring in the roots, the cultivar Bragantina is considered more vulnerable to *F. solani* f. sp. *piperis* in relation to other Amazonian cutivars, and its higher susceptibility may have been determinant for the low expression defense responses, even with the association with arbuscular mycorrhizal fungi. Co-inoculation with two species of AMF (*R. clarus* and *C. etunicatum*) and one pathogen fungi (*F. solani* f. sp. *piperis*) may have resulted in higher plant stress, which was not enough to activate the enzyme systems evaluated in this study.

The production of phenolic compounds can activate the resistance system and provide bioprotection to plants during pathogen stress, and some polyphenols may act as antioxidants [37]. In the present study, the highest phenolic production was observed in the roots of plants inoculated with *F. solani* f. sp. *piperis*; the stress caused by the pathogen fungus may have induced a higher synthesis of phenolics aiming to contain harmful caused by the infection. At 7 dpi, the roots produced more phenolics in AMF + FUS compared to FUS, however at 21 dpi the phenolic content decreased in AMF + FUS, since AMFs may have mitigated the effects of stress by the pathogen in the first days. This decrease in the phenolics may have been caused by the higher severity of the disease at 21 dpi. Another hypothesis to be considered is that the fungi have not colonized the roots sufficiently to induce an effective defense response in the plant, in addition many edaphic microorganisms are antagonistic to pathogenic fungi, but may act differently within the diversity of AM fungal families [38].

The observed decrease in LOX activity may be related to defense suppression events. One study performed the inoculation of *Fusarium oxysporum* f. sp. *phaseoli* in beans (*Phaseolus vulgaris*) showed that the activities of LOX and other oxidative stress-related enzymes decreased during inoculation [39]. Considering that the cultivar Bragantina is susceptible to *F. solani* f. sp. *piperis*, inhibition in the synthesis of LOXs and other enzymes of the jasmonic acid pathway may have facilitated the development and severity of the disease. The VOC production was not detected in the roots, which may be related to the decrease in LOX levels. In addition, *F. solani* f. sp. *piperis* may have inhibited the synthesis of some enzymes and hormones such as jasmonic acid from the LOX pathway in order to facilitate its colonization in the host tissue [40].

Phenylalanine ammonia-lyase is the first enzyme that acts on the phenylpropanoid pathway, and participates in the production of defense substances such as phenolic compounds and phytoalexins [41]. In the present study, the level of PAL in the roots of FUS (14.6; 10.59 U/mL) and AMF + FUS (16.55; 11.47 U/mL) was equivalent, however lower than the CONTROL group (23.8; 21.86 U/mL) at 7 and 21 dpi, respectively. The high degree of stress generated in inoculated and co-inoculated plants may have suppressed PAL activity in the first weeks. *F. solani* f. sp. *piperis* has a hemibiotrophic distinct lifestyle, colonizes the living host initially and subsequently kills and consumes host cells (necrotrophic) [42].

Based on literature reports, the effects observed in PAL activity in co-inoculated plants by AMF and pathogens are distinct. The co-inoculation of *Glomus macrocarpum* and *G. fasciculatum* increased PAL activity in plants of *Lycopersicon esculentum* Mill roots infected by *Fusarium oxysporum* f. sp. *lycopersici* [43]. On the other hand, four AMF formulations made up of three strains containing *Glomus intraradices*, *G. hoi*, *Gigaspora margarita*, or *Scutellospora gigantea* co-inoculated with *Fusarium solani* f. sp. *phaseoli* in bean (*Phaseolus vulgaris* L.) differently induced PAL activity. When tested alone, the consortia of AMFs did not induce PAL activity, however when co-inoculated with the pathogen they induced a high level of PAL, showing great potential for plant defense [12]. In this sense, it is important to perform tests with monospecific inocula and AMF consortia, aiming to select the best AMF species indicated for the biocontrol of plant diseases. A wheat (*Triticum aestivum*) co-inoculation study with *Glomus intradices* AMF and *Gaeumannomyces graminis* cv. Tritici pathogen showed that PAL activity was higher in mycorrhized plants only than in co-inoculated or infected only with the pathogen, suggesting that this enzyme is not induced in wheat compatible interactions [44]. The same condition may have occurred with *P. nigrum*, due to the high susceptibility of the cultivar used.

The hierarchical cluster analyses showed that the co-inoculation maintained the level and composition of volatiles, phenolic compounds, LOX, and PAL enzymes similar to the leaves of the control group, at 7 dpi. After inoculation of AMFs, the establishment of symbiotic colonization may take a few days or weeks, depending on the plant species. A study showed that at 7 dpi, the roots of *P. nigrum* Bragantina cv. were already completely colonized and that the symbionts altered the secondary plant metabolism [11]. Cluster I (Figure 8a) showed that the co-inoculation of *F. solani* f. sp. *piperis* in mycorrhizal plants did not induce sudden changes in plant defense metabolism at 7 dpi. This may have occurred due to the protection that the AMFs can provide against attack by pathogens, as it constitutes a physical barrier to its establishment, due to competition for space [39]. The co-inoculation (Figure 8a), showed a different profile from the control and infected groups at 21 dpi, some constituents analyzed decreased in content. Possibly, as the days went by, the pathogen managed to overcome the barriers imposed by mycorrhizal colonization, which culminated with the development of symptoms of fusariosis in the plants.

The HCA of roots showed that the inoculation maintained the volatile contents similar with the control group, at 7 dpi. *Fusarium solani* f. sp. *piperis* is a fungus that enters the plant through its roots and has a hemibiotrophic lifestyle. This fungus combines the niche of biotrophic and necrotrophic. In the first biotrophic stage, cell death and the host’s immune system are actively suppressed, allowing hyphae to spread throughout the infected plant. Once established in the tissues, the pathogen proceeds to the necrotrophic phase, in which toxins are secreted by the pathogen to induce the death of the host cell [45]. Some effectors of pathogenic fungi are recognized by NOD-like receptors (NLRs) and pattern recognition receptors (PRRs) in the host plant. Biotrophic and hemibiotrophic fungi usually accumulate mutations in these effector genes to escape recognition and prevent the triggering of hypersensitivity responses (HR) [46]. These events may explain this similarity between the defense metabolism of control plants and those inoculated with *F. solani* f. sp *piperis* at 7 dpi (Figure 8b). Finally, the proximity of the infected and co-inoculated groups at 21 dpi shows that co-inoculation failed to activate defense responses efficiently to combat fusariosis.

In general, the co-inoculation of AMFs and *F. solani* f. sp. *piperis* in *P. nigrum* caused negative changes in the defense metabolism of the plant, confirming the severity of fusariosis in black pepper.

## 5. Conclusions

Considering the variations in the secondary metabolites content in FUS and AMF + FUS, with decreases in some volatile compounds and enzymes activities, it is possible the defense of *P. nigrum* Bragantine cv. was suppressed. Thus, it is concluded that infection by *F. solani* f. sp. *piperis* was very severe and *R. clarus* and *C. etunicatum* AMFs were not efficient in promoting the defense of plants in the evaluated days. Even though knowing the potential of AMFs in the biological control of plant diseases and considering the severity of the different strains of *F. solani* f. sp. *piperis*, this work points out the need to carry out new studies with a longer period of time and with other species of AMFs native to the Amazon to evaluate the seedling survival time and changes in secondary metabolism of *P. nigrum,* aiming at the biocontrol of fusariosis.

## Figures and Tables

**Table 1:** Comparison of volatile compounds produced by *Piper nigrum* L. (CONTROL) leaves after inoculation with *Fusarium solani* f. sp. *piperis* (FUS) and co-inoculation with arbuscular mycorrhizal fungi and *F. solani* f. sp. *piperis* (AMF + FUS) (mean ± standard deviation)

Compounds	RI^a^	RI^b^	7 dpi			21 dpi		
			CONTROL	FUS	AMF + FUS	CONTROL	FUS	AMF + FUS
***2E*-hexenal**	**846**	**846**	**17.98 ± 5.20 ^A^**	**6.11 ± 3.77 ^B^**	**1.16 ± 0.92 ^B^**	**2.21 ± 0.58 ^C^**	**3.50 ± 1.68 ^C^**	**3.06 ± 0.83 ^C^**
δ-elemene	1335	1339	2.92 ± 0.14	5.20 ± 0.34	5.15 ± 0.82	4.11 ± 0.18	4.02 ± 0.66	3.42 ± 0.11
α-cubebene	1345	1352	2.70 ± 0.19	4.17 ± 0.77	3.42 ± 0.30	2.45 ± 0.32	2.86 ± 0.14	2.87 ± 0.09
α-copaene	1374	1378	1.95 ± 0.45	6.32 ± 2.68	6.16 ± 0.90	4.11 ± 0.39	3.67 ± 0.34	4.46 ± 0.22
β-cubebene	1387	1392	3.32 ± 0.55	2.93 ± 0.81	0.0	1.08 ± 0.06	0.89 ± 0.03	1.10 ± 0.13
α-gurjunene	1409	1413	0.97 ± 0.23	4.78 ± 2.43	3.94 ± 0.20	3.46 ± 0.14	3.89 ± 0.32	3.62 ± 0.09
β-caryophyllene	1417	1423	2.76 ± 0.63	5.32 ± 1.15	4.73 ± 0.23	4.07 ± 0.33	6.54 ± 1.78	3.80 ± 0.27
β-selinene	1489	1491	1.94 ± 0.49	5.26 ± 0.89	3.86 ± 0.48	4.40 ± 0.28	4.36 ± 0.42	3.57 ± 0.16
*Z*-β-guaiene	1492	1500	0.0	0.0	0.0	6.59	0.0	0.0
viridiflorene	1496	1500	5.36 ± 0.13	5.78 ± 0.88	0.0	0.0	0.0	0.0
bicyclogermacrene	1500	1502	4.60 ± 0.48	4.12 ± 3.15	11.82 ± 4.75	5.20 ± 0.28	4.21 ± 0.79	7.13 ± 2.17
**α-muurolene**	**1500**	**1505**	**1.63 ± 0.32 ^A^**	**2.49 ± 0.63 ^A^**	**3.31 ± 0.62 ^A^**	**16.68 ± 2.44 ^B^**	**0.0 ^C^**	**3.08 ± 0.18 ^D^**
*E-*β-guaiene	1502	1522	0.0	4.77 ± 0.84	0.0	0.0	0.0	0.0
δ-amorphene	1511	1512	0.0	2.18 ± 1.93	0.0	0.0	0.0	0.52 ± 0.12
γ-cadinene	1513	1521	6.78 ± 0.70	8.82 ± 0.40	0.0	0.0	0.0	5.45 ± 0.14
δ-cadinene	1513	1522	3.630.57	3.92 ± 0.39	4.16 ± 0.87	10.60 ± 0.57	10.07 ± 0.55	5.23 ± 0.69
*E*-nerolidol	1561	1567	2.42 ± 0.70	1.11 ± 0.56	1.94 ± 0.79	2.96 ± 0.62	2.08 ± 0.28	1.04 ± 0.12
caryophyllene oxide	1582	1589	1.33 ± 0.29	1.02 ± 0.23	1.50 ± 0.09	2.03 ± 0.26	1.38 ± 0.17	1.52 ± 0.33
α-*epi*-muurolol	1640	1647	3.05 ± 0.84	3.97 ± 0.78	0.0	0.0	0.0	0.0
**α-muurolol**	**1644**	**1651**	**20.59 ± 2.71 ^A^**	**24.52 ± 0.83 ^B^**	**0.0 ^C^**	**0.0 ^D^**	**3.23 ± 0.27 ^E^**	**10.91 ± 0.44 ^F^**
cubenol	1645	1653	0.0	14.20 ± 4.73	18.97 ± 2.02	3.15 ± 0.08	9.07 ± 0.34	1.79
α-cadinol	1652	1659	0.86 ± 0.29	1.44 ± 0.47	0.56 ± 0.06	0.0	0.0	4.39 ± 0.36
Monoterpene hydrocarbons			0.27 ± 0.43	0.0	0.0	0.76 ± 0.09	1.90 ± 0.51	3.64 ± 2.12
Oxygenated monoterpenes			0.72 ± 0.20	1.05 ± 0.58	0.0	1.40 ± 0.22	1.49 ± 0.24	1.59 ± 0.31
Sesquiterpene hydrocarbons			**45.09 ± 7.54 ^A^**	**78.86 ± 20.67 ^A^**	**56.25 ± 10.41 ^A^**	**72.92 ± 6.86 ^A^**	**51.93 ± 6.80 ^A^**	**55.20 ± 6.40 ^A^**
Oxygenated Sesquiterpenes			**29.96 ± 5.70 ^A^**	**50.04 ± 9.39 ^A,B^**	**28.64 ± 4.64 ^A,C^**	**15.35 ± 2.90 ^A^**	**21.69 ± 2.98 ^A^**	**24.63 ± 3.82 ^A^**
Phenylpropanoids			0.0	0.0	0.0	0.0	0.0	0.0
Others			18.36 ± 5.40	6.29 ± 3.83	2.48 ± 1.18	3.24 ± 1.15	4.97 ± 2.01	5.09 ± 1.94
**Total**			**94.40 ± 19.27**	**136.25 ± 34.48**	**87.37 ± 16.23**	**93.67 ± 11.21**	**81.98 ± 12.53**	**90.15 ± 14.58**

This table contains only volatile compounds above 2% present in at least one of the treatments. RI^a^: Retention Index of Library; RI^b^: Retention index calculated; Different letters varied statistically by Tukey’s test (*p* < 0.05).

**Table 2:** Comparison of volatile compounds produced by *Piper nigrum* L. (CONTROL) roots after inoculation with *Fusarium solani* f. sp. *piperis* (FUS) and co-inoculation with arbuscular mycorrhizal fungi and *F. solani* f. sp. *piperis* (AMF + FUS) (mean ± standard deviation)

Compounds	RI^a^	RI^b^	7 dpi			21 dpi		
			CONTROL	FUS	AMF + FUS	CONTROL	FUS	AMF + FUS
n-octane	800	775	0.0	0.0	7.72 ± 3.30	0.0	0.0	0.0
n-nonane	900	900	3.53 ± 0.84	4.02 ± 0.47	3.79 ± 1.61	4.70 ± 0.71	8.24 ± 1.25	7.23 ± 0.95
α-pinene	932	927	2.28 ± 0.41	2.76 ± 0.24	1.75 ± 0.26	3.42 ± 0.76	3.58 ± 0.43	3.44 ± 1.07
canfene	946	945	1.64 ± 0.93	3.95 ± 1.30	1.46 ± 0.53	8.17 ± 0.34	6.36 ± 1.25	4.50 ± 1.95
β-pinene	974	971	4.05 ± 1.38	3.11 ± 0.46	2.16 ± 0.20	3.42 ± 1.10	3.98 ± 0.76	3.71 ± 1.57
**limonene**	**1024**	**1022**	**6.31 ± 1.70**	**4.88 ± 0.69**	**3.44 ± 0.30**	**6.25 ± 1.49**	**5.81 ± 1.64**	**6.53 ± 2.12**
camphor	1141	1146	1.82 ± 0.49	3.17 ± 0.56	0.87 ± 0.46	1.57 ± 0.38	1.36 ± 0.59	0.46 ± 0.14
isoborneol	1155	1158	1.62 ± 0.45	0.54 ± 0.13	0.44 ± 0.19	2.31 ± 0.36	1.60 ± 0.35	0.62 ± 0.10
**δ-elemene**	**1335**	**1337**	**12.68 ± 1.23**	**8.90 ± 1.88**	**10.33 ± 3.50**	**4.06 ± 2.41**	**5.50 ± 2.46**	**2.98 ± 0.92**
**β-caryophyllene**	**1417**	**1423**	**32.93 ± 5.34**	**34.58 ± 2.38**	**42.79 ± 9.36**	**26.60 ± 3.21**	**27.21 ± 1.80**	**26.32 ± 1.16**
Monoterpene hydrocarbons			**15.37 ± 4.86**	**15.74 ± 3.17**	**15.37 ± 4.86**	**22.63 ± 4.19**	**21.10 ± 4.37**	**19.75 ± 7.13**
Oxygenated monoterpenes			3.44 ± 0.95	3.71 ± 0.68	1.31 ± 0.65	3.88 ± 0.74 ^A,B^	2.96 ± 0.93 ^A^	1.08 ± 0.24 ^A,C^
Sesquiterpene hydrocarbons			**51.80 ± 7.81**	**49.61 ± 5.34**	**51.80 ± 7.81**	**35.37 ± 7.23**	**37.07 ± 5.67**	**32.91 ± 3.06**
Oxygenated Sesquiterpenes			2.18 ± 0.84	3.48 ± 0.83	2.42 ± 0.18	2.42 ± 1.12	0.20 ± 0.04	0.94 ± 0.60
Others			3.66 ± 0.91	4.19 ± 0.49	11.62 ± 4.91	5.68 ± 0.84	8.70 ± 1.29	7.61 ± 0.99
**Total**			**76.44 ± 15.37**	**76.74 ± 10.51**	**76.44 ± 15.37**	**69.98 ± 14.11**	**70.03 ± 12.30**	**62.29 ± 12.02**

This table contains only volatile compounds above 2% present in at least one of the treatments. RI^a^: Retention index of library; RI^b^: Retention index calculated; Different letters varied statistically by Tukey’s test (*p* < 0.05).

## Data Availability

The data presented in this study are available on request from the corresponding author.
